# Chemiresistive/SERS dual sensor based on densely packed gold nanoparticles

**DOI:** 10.3762/bjnano.6.259

**Published:** 2015-12-29

**Authors:** Sanda Boca, Cosmin Leordean, Simion Astilean, Cosmin Farcau

**Affiliations:** 1 Nanobiophotonics and Laser Microspectroscopy Center, Interdisciplinary Research Institute on Bio-Nano-Sciences, Babes-Bolyai University, 42 T. Laurian, 400271 Cluj-Napoca, Romania; 2 Faculty of Physics, Babes-Bolyai University, 1 M Kogalniceanu, 400084 Cluj-Napoca, Romania

**Keywords:** colloidal nanoparticles, convective self-assembly, interparticle gaps, surface enhanced Raman scattering, chemiresistor

## Abstract

Chemiresistors are a class of sensitive electrical devices capable of detecting (bio)chemicals by simply monitoring electrical resistance. Sensing based on surface enhanced Raman scattering (SERS) represents a radically different approach, in which molecules are optically detected according to their vibrational spectroscopic fingerprint. Despite different concepts are involved, one can find in the literature examples from both categories reporting sensors made of gold nanoparticles. The same building blocks appear because both sensor classes share a common principle: nanometric interparticle gaps are needed, for electron tunneling in chemiresistors, and for enhancing electromagnetic fields by plasmon coupling in SERS-based sensors. By exploiting such nano-gaps in self-assembled films of gold nanoparticles, we demonstrate the proof of concept of a dual electrical/optical sensor, with both chemiresistive and SERS capabilities. The proposed device is realized by self-assembling 15 nm gold nanoparticles into few micrometers-wide strips across commercially available interdigitated electrodes. The dual-mode operation of the device is demonstrated by the detection of a biologically relevant model analyte, 4-mercaptophenyl boronic acid.

## Introduction

The development of optical sensors is still following an ascendant slope nowadays and ongoing efforts are made to get them more accurate, rapid, portable, and inexpensive. This is mostly due to the promising potential they present in application fields such as health care, food contamination, environmental safety and security, among others [1]. Today, a reliable type of optical sensor must be able to identify and quantify the investigated analyte, ideally by the use of a low-cost chip made by simple manufacturing procedures. Noble metal nanoparticles (NPs) demonstrated repeatedly their effectiveness as transducing elements in optical sensors based on surface plasmon resonance, surface-enhanced fluorescence or surface-enhanced Raman spectroscopy (SERS) [2–3]. Among these analytical techniques, SERS is particularly interesting because it can specifically identify the analyte by the unique vibrational signature of chemical groups.

Another class of promising sensors are chemiresistors, which are sensitive electrical devices capable of detecting (bio)chemicals by simply monitoring electrical resistance. They require simple DC circuitry, and are therefore ideal for developing wearable/portable devices for protection from chemical exposure, or for environmental monitoring. Assemblies of gold NPs were recently proposed as chemiresistor sensors, exhibiting reliable responses to the analyzed compounds [4]*.* In most cases very small particles were used (3–7 nm), functionalized with specific ligands that facilitate their assembly into thin nanoparticle films through cross-linking reactions, and connected to (micro)electrodes [5–6].

In this work we propose a dual electrical/optical sensor (DEOS) capable of exploiting both chemiresistive and SERS-based sensing. The device consists of a self-assembled film of spherical gold nanoparticles that is electrically connected by interdigitated electrodes (IDE) on a commercially available chip. We tested the electrical response of the sensor to external stimuli and validated its capability to detect the model analyte mercaptophenyl boronic acid. In contrast with previous nanoparticle-based chemiresistors, which use small nanoparticles (3–7 nm), we used larger gold nanoparticles (15 nm) in the sensor design, which allow one to obtain useful SERS measurements at the same time. Thus, we were able to optically detect and identify the analyte by its vibrational SERS signature. To our knowledge this is the first demonstration of a dual electrical/optical sensing concept based on gold nanoparticles, combining unique advantages of both chemiresistor and SERS sensors.

## Results and Discussion

### Dual sensing concept

The main idea of this work stems from the fact that the same physical systems (noble metal colloids) and the same fabrication tools (colloidal self-assembly) could in principle yield novel nano-enabled devices with both optical and electrical functionalities.

**Figure 1:**
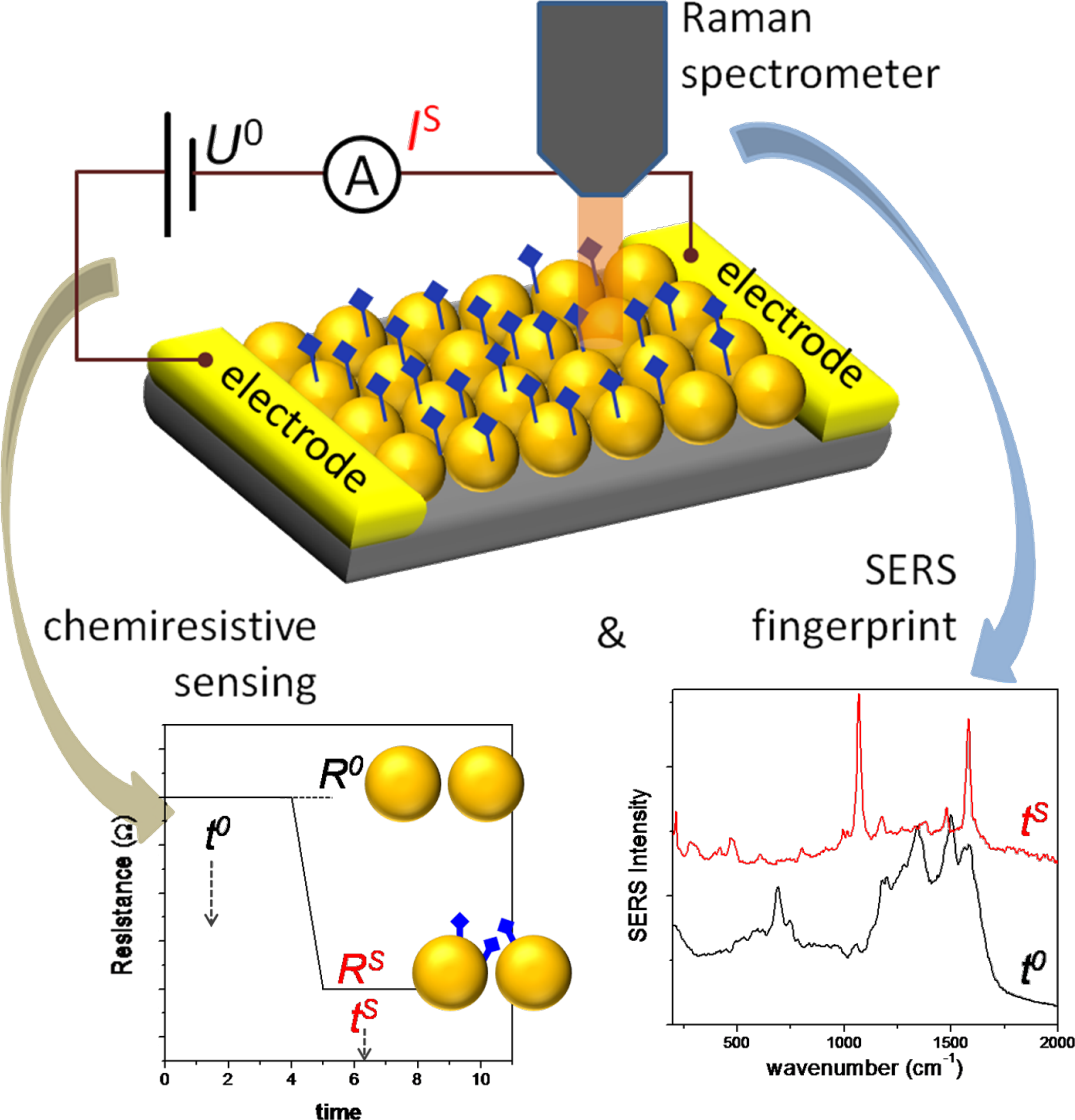
Schematic representation of the concept of a dual electric/optical sensor made of a nanoparticle assembly connected by metal electrodes on a substrate.

A graphical summary describing the envisaged DEOS concept is given in Figure 1. An array of noble metal colloids assembled between electrodes on a solid substrate can lead to electrical charge flow through the nanoparticle assembly by applying a voltage on the electrodes. This system can work as a resistive sensor based on the dependence of tunneling currents on the inter-NP tunnel barrier, which can be affected by the molecular species adsorbed on the NPs, or by changes of the inter-particle distance. Plasmon coupling and SERS enhancement are also known to strongly depend on inter-particle nanoscale gaps. Thus, a dense particle organization and the molecular capping layer (preventing metal particles from touching each other) control both SERS enhancement and the electrical resistance of the particle assembly. External molecular species adsorbed on the nanoparticles can modify the electrical response of the film, and can also be identified by their SERS spectrum (graphs and spectra are just for illustration). The advantages offered by such a dual sensor would be: an electrical readout, which is always preferred since it is the most simple and inexpensive method to implement in a final product/device; complementary, by optics (vibrational spectroscopy, SERS), specific information can be retrieved on the identity of the analyte. Such a DEOS would thus combine unique advantages of both electric and optical sensors.

### Colloidal gold nanoparticles as building blocks

A request for a good control of the NP assembling process is represented by the chemical stability of the used colloid. We observed that by increasing the concentration of the colloid, this stability decreases proportionally. Hence, to increase the stability of the concentrated collidal solution, the originally citrate-capped AuNPs were functionalized with folic acid molecules by electrostatic interaction between the negative surface of the particles and the positive amino groups of folic acid molecules. Folic acid is a low molecular weight vitamin compound, which has been shown to be an effective targeting vector of various cancer cell lines which over-express folate receptors [7]. It also proved to be an effective capping ligand for linking onto various polymer backbones or biological molecules. Optical absorption spectra of the colloidal suspension recorded before and after incubation with folic acid confirmed the capping of AuNPs (redshift of the dipolar plasmon band) (Figure S1, Supporting Information File 1). DLS measurements correlate with the optical absorption results: for FA-capped nanoparticles (21 nm) the obtained hydrodynamic diameter was larger than for bare colloidal nanoparticles (18 nm) (Figure S2, Supporting Information File 1). Zeta potential measurements of the concentrated suspension show an average value of ζ = −32 mV, which prove a good electrostatic stability of the folic acid-capped AuNPs in the aqueous system [8].

#### Assembly of gold nanoparticles on IDE

In the conventional way, convective self-assembly (CSA) was used for growing thin colloidal films on planar substrates, by particle deposition from a colloidal suspension along the substrate–liquid–air triple contact line (called also meniscus) [9–10]. Further, the technique was refined in order to allow for the fabrication of parallel strips of nanoparticle films on various substrates [11]. One such variant was named stop&go convective self-assembly (SG-CSA) [12]. If up to now SG-CSA was employed only on planar solid substrates like glass slides [13] or PET films [12], in the present work, we used as substrate commercial IDE. We chose here SG-CSA and not a simpler variant because it is neccessary for the assembly proccess. It is known that topography on a substrate strongly influences the assembly of nanoparticles, especially when the NPs are smaller than those topographical features. SG-CSA allows to restrict the meniscus movement at will, by imposing (through computer-controlled software) a periodic movement and stopping of the substrate. During the 'stop' phase the particles are accumulated at the meniscus and deposited on the substrate. During the 'go' phase, the meniscus is rapidly translated to the next stop position. Our setup also imposes a straight triple contact line, which ensures deposition of strips of desired geometry. Furthermore, the gold electrodes are positioned perpendicular to the meniscus, and parallel to the translation direction. This ensures that during the 'go' phase no undesired/uncontrolled pinning by the electrodes occurs.

#### Sensor characterization

**Figure 2:**
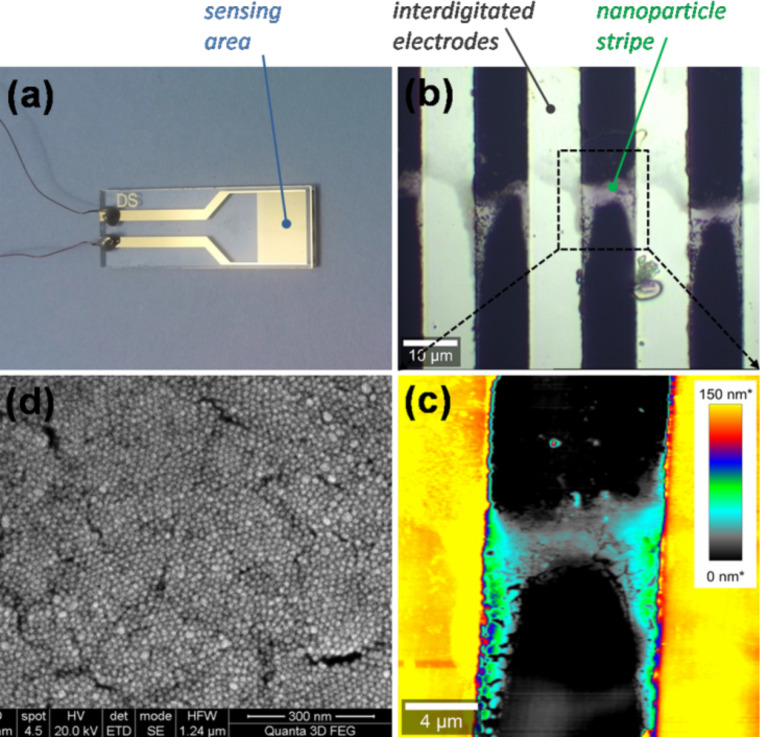
(a) Optical image of the IDE (Dropsens) electrically connected by conductor wires; (b) Optical image of a region containing Au NPs assembled as a strip between a pair of Au IDE. (c) AFM image of the region shown in b); (d) SEM image showing the dense NP assembly.

An optical image of the commercial IDE chip is presented in Figure 2a. As better seen in Figure 2b it consists of 10 µm wide gold electrodes spaced by 10 µm. Their height is 150 nm. Strips made of gold nanoparticles are deposited through SG-CSA, perpendicular to the IDE with the purpose of electrically connecting them. Five gold nanoparticle strips were fabricated, spaced by 100 µm. The optical microscopy image presented in Figure 2b displays the IDE gold electrodes that are connected by gold nanoparticle strips. The zoom in AFM image (Figure 2c) shows that the gold nanoparticle strips adopted an elongated shape near the gold electrodes, in agreement with the fact that the meniscus is deformed by topographical features during CSA. Finally, the SEM image in Figure 2d indicates that the gold nanoparticles in the strips are assembled with a high density packing. This is due to the good stability of the colloid during assembly, achieved by capping NPs with FA. The current–voltage (*I*–*V*) behavior of the assembled films was linear in the measured range, indicating an ohmic behavior (Figure S3, Supporting Information File 1).

#### Dual-mode sensing

**Figure 3:**
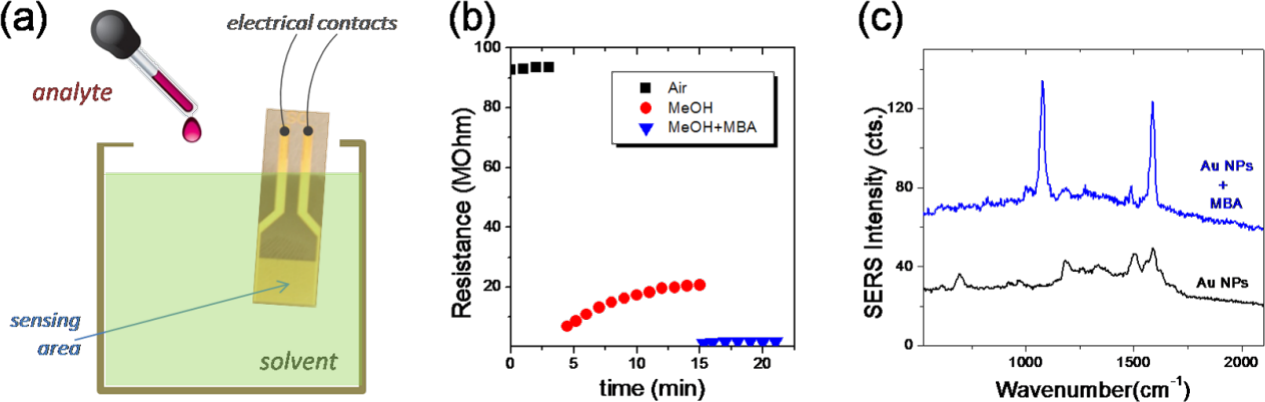
(a) Scheme of the experimental setup for chemiresistive detection; (b) Chemiresistor response: electrical resistance as function of time, as the sensor is exposed to different media; (c) SERS spectra on the chemiresistor surface before and after exposure to MBA.

Figure 3 presents results on the dual-sensing capabilities of the developed nanoparticle-based DEOS. The experimental setup for the resistive sensing is depicted in Figure 3a. The electrically connected IDE with assembled Au NPs is immersed in a methanol (MeOH) bath, by taking care to leave the two electrode tracks outside of the liquid. Since the Au NPs are dispersable in water, the exposure to alcohol does not damage markedly the assembled film.

Upon immersion of the sensor from air into MeOH a large and instant drop of the resistance was observed, from about 93 MΩ to about 7 MΩ for the sample discussed here. The resistance slowly increases, and stabilizes around 20 MΩ after 8–10 min. This behavior could be interpreted as an accommodation of the NP assembly into the solvent environment; at the same time it could indicate small disturbances in the microscopic NP arrangement, induced by the solvent. Subsequently, the analyte (here MBA in methanol solution) is pipetted (one drop) into the methanol bath (the final MBA solution concentration is 10^−5^ M). Again, the resistance of the NP assembly suddenly drops, to 1.3 MΩ and stabilizes at 1.7 MΩ within 1–2 min. This change in resistance represents a drop of more than 90% from the value it had before adding the MBA analyte. Qualitatively, we observed the same behavior across several samples, although the resistance values and change of resistance are not exactly the same. Clearly, sample resistance depends on the precise geometry of the NP strips that connect the IDEs. For the resistance change, values between 65 and 90% were observed. The resistance drop upon adsorption of the analyte can be understood by considering that charge transport through the NP assembly occurs mainly by electron tunneling. The tunneling resistance depends exponentially on the interparticle distance *l* (surface to surface): *R*_T_ ≈ e^β^*^l^*, where β is a tunneling decay constant that describes the tunneling of electrons along the organic capping molecules. By changing the molecular content of the interparticle gap both *l* and β can be expected to suffer modifications, which translate into changes of the macroscopic resistance. A decrease of the resistance upon analyte sorption has been already observed in chemiresistors based on rigidly cross-linked nanoparticle films [6,14]. The effective permittivity in the environment of the nanoparticles can increase due to the adsorption of analytes, which can replace ligands or fill up inter-molecular voids on the surface of the nanoparticles. This can decrease the activation energy for electron transfer, which is in turn observed as a resistance decrease.

Moreover, in our system MBA is a thiol-terminated molecule while folic acid is not, and therefore one can expect that the MBA molecules can replace folic acid ligands from the surface of the gold particles. MBA is smaller than folic acid, and this observation is another argument sustaining the above discussion.

Afterwards, the sample was removed from the liquid environment, dried, and SERS spectra were measured. Figure 3c presents the SERS spectra recorded on the NP wires at the beginning of the experiment, i.e., before adsorbing the analyte, and after MBA adsorption, after the chemiresistor experiment. After MBA exposure, two intense vibrational bands are observed at 1078 and 1587 cm^−1^. These bands are assigned to the benzene ring, specifically being attributed to β_CCC_ + ν_CS_ and ν_CC_ with β and ν denoting bending and stretching modes, respectively [15–16]. This is an indication for the adsorption of MBA molecules. These bands were observed also in control experiments performed on another kind of gold SERS substrate without capping ligands such as folic acid on the NPs (see Supporting Information File 1). Some less intense bands, e.g., at 1182 and 1487 cm^−1^, can also be observed, which were present in the spectrum measured before MBA exposure, due to folic acid molecules. Note that a flat gold film deposited by thermal evaporation did not exhibit any visible Raman bands, under the same experimental conditions (exposure to methanol, MBA solution, drying). The SERS enhancement factor, EF, for this kind of self-assembled Au nanoparticle films is on the order 10^6^ to 10^7^, as we previously demonstrated in similar systems. It was calculated using the equation:


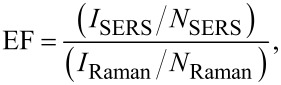


where *I*_SERS_ is the SERS intensity, *I*_Raman_ is the Raman intensity, *N*_SERS_ is the number of probed molecules in SERS, and *N*_Raman_ is the number of probed molecules in Raman measurements [17]. Further studies about the sensitivity and selectivity of the DEOS are required too fully ascribe these on the quantitative level, especially as a comparative assessment of the electric and the optical sensing approaches. Finally, although the measurements reported above are done sequentially, and not simultaneous, one can easily accept the fact that the progress to a simultaneous experiment is only a technical matter; it requires technologies such as PDMS molding of a fluidic chamber, and online analyte injection, which we might tackle in the near future.

## Conclusion

This work demonstrated the proof-of-concept of a nanoparticle-based dual-mode (bio)chemo-sensor capable of working as both chemiresistor and SERS substrate. At the heart of the proposed DEOS lie strips of gold nanoparticles formed by convective self-assembly, without implying lithographic procedures. The NP strips are fabricated directly across interdigitated electrodes on commercially available chips, making the DEOS easy to handle, and compatible with existing electrochemistry equipment. The adsorption of MBA model molecular analyte on the nanoparticles caused a change of electrical resistance, proving the chemiresistor ‘half’ of the DEOS. By subsequent SERS analyses information on the identity of the species affecting the resistance was obtained through its specific vibrational Raman bands. Further development is necessary, e.g., by introducing molecular recognition elements, in order to improve the selectivity of the sensor. Knowledge and technology existing on chemiresistive biosensors on one side and SERS detection on the other, can be commonly exploited for developing advanced dual sensors benefiting from the advantages of the two separated, up to now, research fields. For example, by integration with microfluidics technology, the simultaneous operation in both chemiresistor and SERS-based modes should readily become possible. We expect that this innovative type of dual sensor will combine and multiply the benefits offered by its two facets, electric and optic, which were previously only separately exploited for (bio)sensing.

## Experimental

**Materials and reagents:** Hydrogen tetrachloroaurate(III) trihydrate (HAuCl_4_·3H_2_O), trisodium citrate (C_6_H_5_Na_3_O_7_), 4-mercaptophenylboronic acid 90% (HSC_6_H_4_B(OH)_2_), folic acid (C_19_H_19_N_7_O_6_) were purchased from Sigma-Aldrich. Gold interdigitated electrodes (IDE) were purchased from Dropsens.

**Gold nanoparticle synthesis and stabilization:** Colloidal gold nanoparticles (AuNPs) were synthesized by the aqueous reduction of HAuCl_4_ with trisodium citrate. An amount of 100 mL of 10^−3^ M HAuCl_4_·3H_2_O was boiled. A solution of 38.8·10^−3^ M sodium citrate (10 mL) was added under stirring. After the color changed from yellow to burgundy-red, the heat was stopped, and stirring continued until the colloid reached room temperature. Folic acid was mixed with the colloidal nanoparticles and stirred overnight at RT. The capped AuNPs were purified and concentrated by centrifugation.

**Convective self-assembly of gold nanoparticles on IDE:** Commercial IDE were used as platforms for NP assembly. These were washed in ethanol, water and dried. An UV–ozone treatment (NovaScan PSDP-UVT) was performed to increase the hydrophilicity of the IDE surface. The convective self-assembly (CSA) deposition setup consists in a glass deposition plate placed at an angle of 30° over the horizontal IDE fixed substrate. A drop (5 µL) of gold nanoparticles was placed in the wedge between the plate and IDE. Strip-like assemblies of gold NPs are formed by computer-controlled periodic movement and stopping of the substrate. The process is performed under ambient conditions at 22–25 °C and a humidity of 32–35%.

**Sample characterization:** Optical extinction spectra were measured with a Jasco V-670 spectrophotometer. Particle morphology was determined by transmission electron microscopy (TEM) using a JEOL JEM1010 microscope. Zeta potential and hydrodynamic diameter of the particles were determined by using a Zetasizer Nano-ZS90 (Malvern Instruments). SERS spectra were recorded with a confocal Raman microscope (Witec alpha300 R) using a 100× objective (NA = 0.9) and 785 nm laser. Topography was studied by intermittent contact mode AFM measurements using the same Witec system. Electrical measurements were performed with a Keithley electrometer; a voltage of 1 V was applied in the chemiresistor experiments.

## Supporting Information

Supporting Information provides optical extinction and DLS characterization of the gold colloid, current–voltage characteristics of the gold nanoparticle strips, and SERS of MBA on AuFoN substrate.

File 1 Additional experimental data.
