# Supplementary material for: Chemiresistive/SERS dual sensor based on densely packed gold nanoparticles
Source: Beilstein J Nanotechnol. 2015 Dec 29;6:2498–503. doi: 10.3762/bjnano.6.259 (PMC4734427; doi:10.3762/bjnano.6.259)
Supplement: File 1 — Additional experimental data. [file Beilstein_J_Nanotechnol-06-2498-s001.pdf]

Supporting Information

for

Chemiresistive/SERS dual sensor based on densely packed gold nanoparticles

Sanda Boca[1], Cosmin Leordean[1], Simion Astilean[1,2] and Cosmin Farcau*[1]

Address: [1]Nanobiophotonics and Laser Microspectroscopy Center, Interdisciplinary Research Institute on Bio-Nano-Sciences, Babes-Bolyai University, 42 T. Laurian, 400271 Cluj-Napoca, Romania and [2]Faculty of Physics, Babes-Bolyai University, 1 M Kogalniceanu, 400084 Cluj-Napoca, Romania

Email: Cosmin Farcau - cosmin.farcau@phys.ubbcluj.ro

* Corresponding author

Additional experimental data

Figures S1 and Figure S2 present extinction spectra and DLS graphs, respectively, on the gold colloid before (bare) and after (FA capped) capping of the NPs with folic acid molecules.

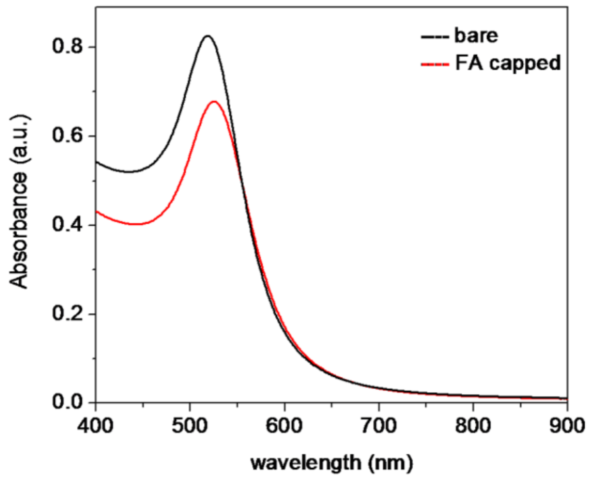

Figure S1: Optical absorption spectra of colloidal gold nanoparticles before (black curve) and after capping with folic acid molecules (red curve).

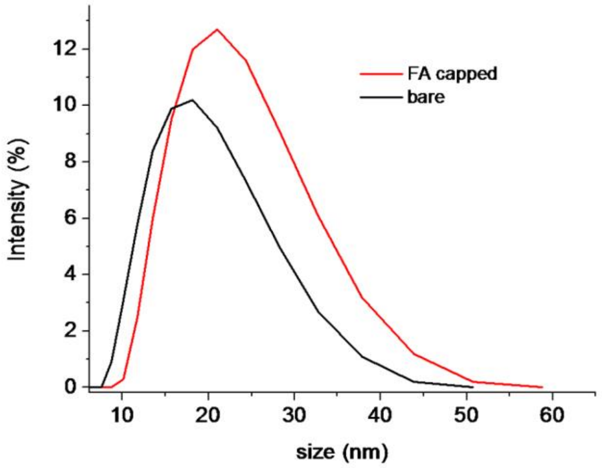

Figure S2: Size distribution obtained by DLS, on colloidal gold nanoparticles before (black curve) and after capping with folic acid molecules (red curve).

A representative current–voltage (*I–V*) dependence curve is displayed in Figure S3. As can be observed a linear behavior is manifested by the assembled NP strips.

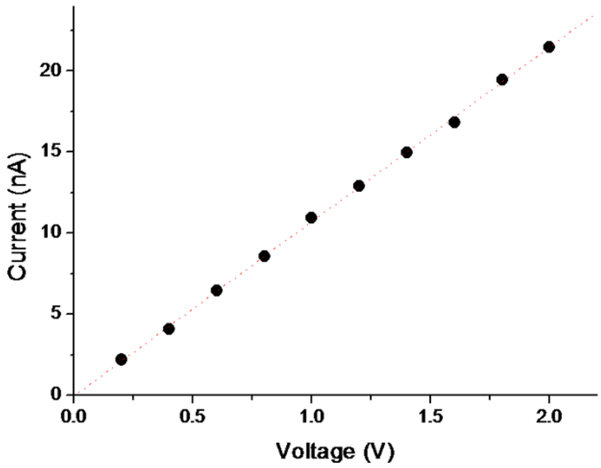

Figure S3: *I–V* dependence of Au NP strips.

SERS spectra of mercaptophenyl boronic acid (MBA) adsorbed on the AuNP strips is compared to SERS of MBA adsorbed on Au film over nanospheres (AuFoN) substrates. In contrast with chemically synthesized AuNPs, the AuFoNs have a clean gold surface (these are obtained by Au evaporation, without other chemicals). The similarity of the two spectra ensures that the spectra obtained on the dual sensor can indeed be reliably attributed to MBA.

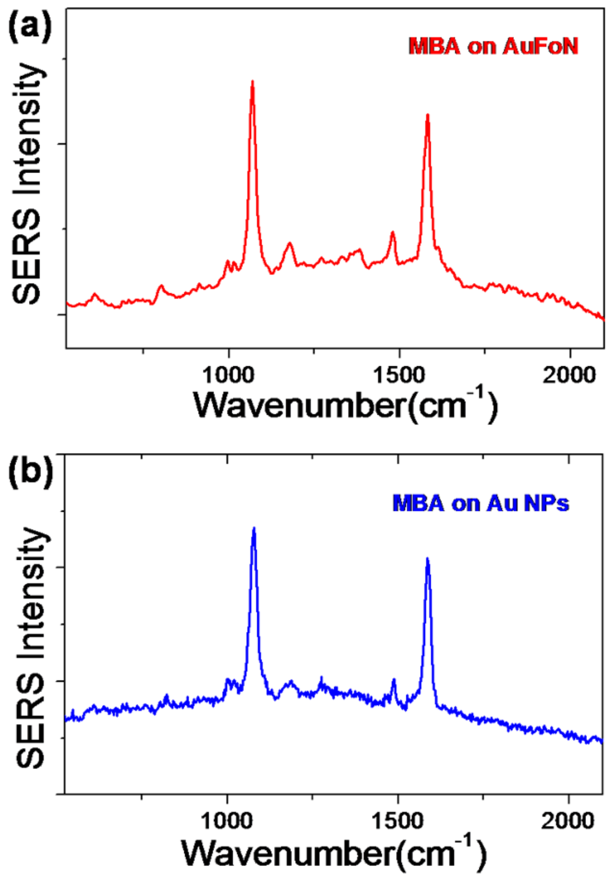

Figure S4: SERS spectra of MBA: (a) on AuFoN substrate; (b) on AuNP strip.
